# First-trimester hemoglobin, haptoglobin genotype, and risk of gestational diabetes mellitus in a retrospective study among Chinese pregnant women

**DOI:** 10.1038/s41387-024-00309-y

**Published:** 2024-06-29

**Authors:** Yue Li, Fang Wang, Xinmei Huang, Shuhang Zong, Yi Shen, Lina Guo, Qiongyi Cai, Tiange Sun, Rui Zhang, Zhiyan Yu, Liwen Zhang, Shufei Zang, Jun Liu

**Affiliations:** 1 Department of Endocrinology, Shanghai Fifth People’s Hospital, Fudan University, 801 Heqing Road, 200240 Shanghai, China; 2 Department of Obstetrics and Gynecology, Shanghai Fifth People’s Hospital, Fudan University, 801 Heqing Road, 200240 Shanghai, China

**Keywords:** Gestational diabetes, Risk factors

## Abstract

**Background:**

This study aimed to assess whether the Haptoglobin (Hp) genotype influences the relationship between hemoglobin (Hb) levels and the development of gestational diabetes mellitus (GDM). Additionally, it sought to evaluate the interaction and joint association of Hb levels and Hp genotype with GDM risk.

**Methods:**

This retrospective study involved 358 women with GDM and 1324 women with normal glucose tolerance (NGT). Peripheral blood leukocytes were collected from 360 individuals at 14–16 weeks’ gestation for Hp genotyping. GDM was diagnosed between 24–28 weeks’ gestation. Interactive moderating effect, joint analysis, and mediation analysis were performed to evaluate the crosslink of Hb levels and Hp genotype with GDM risk.

**Results:**

Women who developed GDM had significantly higher Hb levels throughout pregnancy compared to those with NGT. Increase first-trimester Hb concentration was associated with a progressive rise in GDM incidence, glucose levels, glycosylated hemoglobin levels, Homeostasis Model Assessment for Insulin Resistance (HOMA-IR) values, cesarean delivery rates, and composite neonatal outcomes. Spline regression showed a significant linear association of GDM incidence with continuous first-trimester Hb level when the latter exceeded 122 g/L. Increased first-trimester Hb concentration was an independent risk factor for GDM development after adjusting for potential confounding factors in both the overall population and a matched case-control group. The Hp2–2 genotype was more prevalent among pregnant women with GDM when first-trimester Hb exceeded 122 g/L. Significant multiplicative and additive interactions were identified between Hb levels and Hp genotype for GDM risk, adjusted for age and pre-pregnancy BMI. The odds ratio (OR) for GDM development increased incrementally when stratified by Hb levels and Hp genotype. Moreover, first-trimester Hb level partially mediated the association between Hp genotype and GDM risk.

**Conclusion:**

Increased first-trimester Hb levels were closely associated with the development of GDM and adverse pregnancy outcomes, with this association moderated by the Hp2–2 genotype.

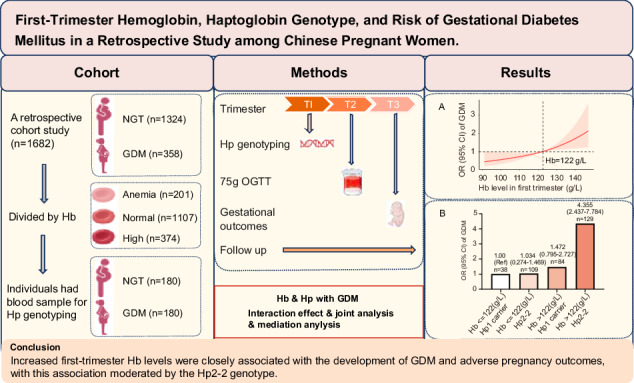

## Background

Gestational diabetes mellitus (GDM) is diagnosed as diabetes manifesting in the second or third trimester of pregnancy, absent prior overt diabetes [1]. The prevalence of GDM has been increasing worldwide, affecting 1% to over 30% of pregnancies, in parallel with the worldwide epidemic of obesity and diabetes [2]. GDM confers significant maternal and fetal risks, including increased likelihood of cesarean section, preterm delivery, preeclampsia, macrosomia, neonatal hypoglycemia, hyperbilirubinemia, respiratory distress syndrome, and need for admission to a neonatal intensive care unit [3]. Established risk factors for GDM include maternal overweight and obesity, advanced maternal age, previous GDM, and family history of type 2 diabetes mellitus, although its exact pathophysiology remains poorly understood.

Hemoglobin (Hb), an indicator of nutritional status, is routinely tested at a perinatal visit. Several studies have shown that high Hb level and iron supplementation with good Hb status during pregnancy are associated with many pregnancy complications [4, 5]. The majority of studies have reported that high Hb level in the first trimester increases the risk of GDM development [4, 6], while a study in Iran reported that higher Hb level in the second trimester was associated with increased risk for GDM development [7]. Nonetheless other studies reported no such association [8, 9]. Studies have yet to define high maternal Hb level according to quartiles or median [4, 6, 10] and there remain questions about what specific Hb threshold can predict GDM risk or be protective for both mother and child.

Previous studies have shown that increased Hb levels are associated with insulin resistance and impaired beta-cell function [11]. As the primary iron reservoir in the body, Hb contributes to elevated iron stores, leading to the production of reactive oxygen species and oxidative stress, which damage pancreatic beta cells and reduce insulin synthesis and secretion [12]. Haptoglobin (Hp) binds cell-free Hb, facilitating its removal from circulation via the CD163 monocyte receptor [13]. There are two Hp alleles, 1 and 2, forming three genotypes (Hp1-1, Hp2-1, and Hp2-2). The Hp2-2 genotype produces a larger, more cyclic Hp protein that less effectively binds and clears free Hb, thus reducing its antioxidative capacity [14]. Previous studies have shown that individuals with an Hp2-2 genotype were more likely to develop GDM [15]. Due to their close functional relationship, it is unclear whether the effect of Hb on GDM development is regulated by Hp genotype.

This clinical study is the first to confirm the association between Hb levels and GDM as well as adverse pregnancy outcomes. The distribution of Hp genotypes was tested in both women with healthy pregnancies and those with GDM. In addition, interactive moderating effect, joint analysis, and mediation analysis were conducted to evaluate the interaction between Hb concentration and Hp genotype, and their combined influence on the development of GDM.

## Methods

### Study population, inclusion, and exclusion criteria

**Fig. 1:**
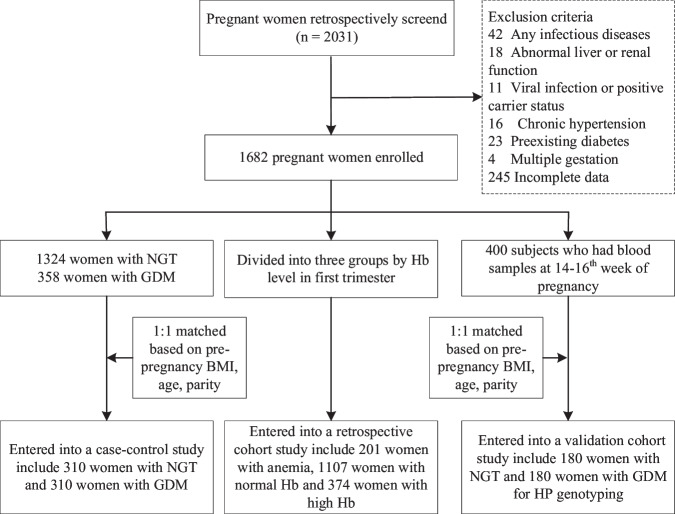
Flowchart of the study.

This retrospective study included 2031 women who underwent prenatal examinations and subsequent normal delivery from January 2016 to January 2022 at the GDM care center of the Fifth People’s Hospital of Shanghai, Fudan University and the Department of Obstetrics at Wujing Hospital in Minhang District, Shanghai. The retrospective analysis followed the procedure described in Fig. 1.

Women were excluded from the study if they had any of the following: (1) infectious disease within 2 weeks prior to blood cell count; (2) abnormal liver or renal function; (3) viral infection or positive carrier status (hepatitis B virus, syphilis, and HIV); (4) preexisting diabetes; (5) chronic hypertension; (6) multiple gestation; (7) malignant tumor; or (8) preexisting pancreatic exocrine disease. After exclusions, 1682 women (1324 with normal glucose tolerance (NGT) and 358 with GDM) were enrolled in the study.

The criteria for diagnosis of GDM were based on the 2016 American Diabetes Association (ADA) guidelines [1]: fasting blood glucose (FBG) ≥ 5.1 mmol/L and 1 h BG ≥ 10.0 mmol/L or 2 h BG ≥ 8.5 mmol/L. The study protocol was approved by the Ethics Committee of the Shanghai Fifth People’s Hospital, Fudan University, and all participants provided written informed consent. The study was conducted in accordance with the Declaration of Helsinki.

### Data collection and laboratory assessments during pregnancy

At the initial visit, gestational age was calculated based on the date of last menstruation and confirmed by ultrasonography. At 14-16 weeks’ gestation, after an overnight fast for 12 h, blood samples were collected for blood cell count (XN9000 Automatic Blood Cell Analyzer; Sysmex, Kobe, Japan) and measurement of biochemical parameters (Cobas 8000 Automatic Biochemical Analyzer; Roche, Basel, Switzerland). Among the 1682 women, 400 had blood samples available for subsequent HP genotyping. Blood pressure and anthropometric parameters were recorded and a patient questionnaire was completed. The questionnaire obtained information about last menstruation, method of conception, parity, obstetric history, family history of diabetes, previous history of GDM, iron supplement history, and pre-pregnancy weight. A 75-g oral glucose tolerance test (OGTT) was administered between the 24th and 28th week of gestation after an overnight fast of at least 8 h to all subjects without overt diabetes or GDM in early pregnancy. After delivery, details including gestational age at delivery, mode of delivery, newborn weight, sex of the neonate, and delivery complications were recorded.

### Maternal, delivery and neonatal outcomes

Maternal outcomes included the proportion of participants who experienced excessive gestational weight gain (EGWG), required insulin therapy, or developed hypertensive disorders of pregnancy, such as nonproteinuric pregnancy-induced hypertension, preeclampsia, or eclampsia. Gestational weight gain (GWG) was calculated by subtracting the initial recorded weight at or before 14 weeks’ gestation from the most recent weight measured at the hospital clinic or ward before delivery. EGWG was determined according to the 2009 Institute of Medicine guidelines when GWG exceeded that recommended for the relevant pre-pregnancy body mass index (BMI) category by gestational age at delivery [16]. Delivery outcomes included delivery time, the need for cesarean section, preterm delivery before 37 weeks of gestation, birth length, newborn weight, macrosomia (birth weight >4 kg), large for gestational age (LGA), small for gestational age (SGA), and Apgar score <7 at 1 and 5 min. Infants classified as LGA and SGA were those birth weight were above the 90th percentile or below the 10th percentile, respectively, for gestational age and sex, based on Chinese neonatal anthropometric charts [17]. Neonatal complications included the presence of neonatal hypoglycemia (capillary blood glucose <2.6 mmol/L within 24 h of birth), hyperbilirubinemia (diagnosed by the attending pediatrician), respiratory distress, and neonatal intensive care unit (NICU) admission within 24 h of birth. A composite measure of neonatal complications defined by the presence of any one of these conditions was presented [18].

### Calculation of BMI, HOMA-IR, HOMA-β

BMI was calculated by dividing maternal weight in kilograms by the squared height in meters. Primary methods to evaluate insulin resistance and pancreatic β cell function were as follows: (1) homeostasis model assessment of insulin resistance index (HOMA-IR) = FBG (mmol/L) * fasting insulin (mIU/L)/22.5; and (2) homeostasis model assessment of pancreatic β cell function index (HOMA-β) = 20* fasting insulin (mIU/L)/*[FBG (mmol/L) - 3.5].

### Intervention for GDM

The therapeutic regimen commenced as soon as an individual was diagnosed with GDM. Lifestyle interventions were first initiated followed by the addition of insulin therapy if the goals of glycemic control were not reached (fasting glucose, 5.3 mmol/L, 1-h postprandial glucose, 7.8 mmol/L, or 2-h postprandial glucose, 6.7 mmol/L).

### Hp genotyping

Genomic DNA was extracted from peripheral blood leukocytes collected at 14–16 weeks’ gestation from 180 women with NGT and 180 women with GDM, who were 1:1 case-control matched based on pre-pregnancy BMI, age, and parity from a pool of 400 subjects, using a QIAamp DNA Blood Kit (Qiagen). The Hp genotype was determined by PCR and agarose gel electrophoresis as previously described [19], as detailed in the supplementary methods and shown in Supplementary Fig. S1.

### Statistical analysis

To minimize potential bias from uneven covariates distribution between women with NGT and those with GDM, a 1:1 case-control matching method was employed. Variables matched included pre-pregnancy BMI, age, and parity, with the tolerance of 0.5, 2, and 0, respectively. To further validate the association of Hb status with GDM development and pregnancy outcomes, all subjects were divided into three groups according to Hb level: anemia (<110 g/L), normal Hb (≥110 and ≤130 g/L) and high Hb (>130 g/L). Anemia was defined according to World Health Organization criteria [20], while high Hb level was based on previous studies in the Asian population [10].

Descriptive statistics for the studied variables are presented as mean ± standard deviation (SD) for normally distributed variables, median (interquartile range [IQR]) for non-normally distributed variables, and frequency (percentage) for categorical variables. ANOVA and the Student t-test were used to identify the difference in means between groups, with Bonferroni correction applied for multiple comparisons. Non-normally distributed variables were analyzed using Kruskal-Wallis one-way ANOVA or Wilcoxon tests. Categorical variables were compared using the Chi-square test. HOMA-IR and HOMA-β were log-transformed previously for t-tests or ANOVA. Pearson correlation was used to analyze the correlation between first-trimester Hb level and FBG, 1-h BG, 2-h BG, HOMA-IR, HOMA-β during OGTT. Additionally, we used a random-effects restricted cubic spline model with three knots to test for potential nonlinearity in the association of first-trimester Hb level with GDM incidence.

To determine whether Hb status or Hp genotype was an independent risk factor, logistic regression analysis was performed with GDM classified in a binary manner (present or absent) as the dependent variable. Hb group or Hp genotype, along with traditional or potential confounding factors—including age, pre-pregnancy BMI, neutrophil count, platelet count, TG, and creatinine level in the first trimester - were identified as possible risk factors and included in the logistic regression analysis. Receiver Operating Characteristic (ROC) curves were constructed by Hb concentration, neutrophil count, or platelet count combined with basal factors (age, pre-pregnancy BMI, TG, and FBG in the first trimester) to predict GDM.

To quantify additive and multiplicative interactions, we also included a product term of Hb and Hp in the logistic regression model constructed by Hb, Hp, age, and pre-pregnancy BMI. The odds ratio (OR) with its 95% confidence interval (CI) for the product term was used to measure interaction on the multiplicative scale. The relative excess risk due to interaction (RERI) and corresponding 95% CI were used to measure interaction on the additive scale [21]. To assess the joint associations, we further classified participants into four groups according to Hb level and Hp genotype and estimated OR of GDM risk in different groups. Analysis of the degree of mediation was performed to demonstrate the effect of first-trimester Hb level on the association between Hp genotype and GDM risk. The main parameter was the proportion of mediation, calculated as (indirect effect / total effect) ×100%.

All data were analyzed using SPSS 24.0 software (IBM, Armonk, NY) and R software (version R 4.3.1). A two-tailed *P* < 0.05 value was considered statistically significant.

## Results

### Characteristics of women with NGT and with GDM across all subjects and in the matched case-control study

**Fig. 2:**
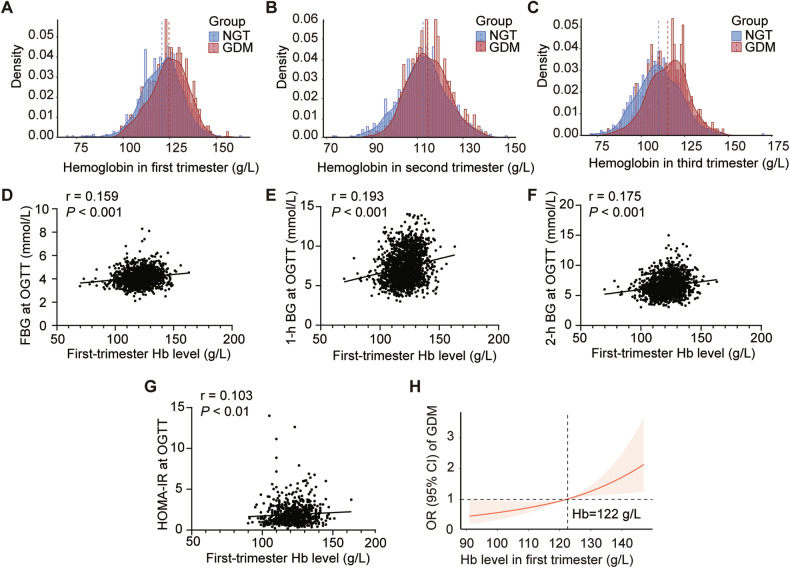
Hemoglobin concentration distribution and correlation with glucose metrics during pregnancy. Histograms of the distribution of Hb concentration throughout pregnancy for all subjects with NGT and with GDM, in first trimester (**A**), in second trimester (**B**) and in third trimester (**C**). Data are presented as mean ± SD and are analyzed by independent sample t test. Spearman correlation analysis of first-trimester Hb level with FBG (**D**), 1-h BG (**E**), 2-h BG (**F**), HOMA-IR (**G**) during OGTT. Continuous association of Hb level in the first trimester with the incidence of GDM, adjusted for GDM history, age and pre-pregnancy BMI (**H**). The OR is expressed per absolute increase in 10 g/L in the Hb value at baseline. The shaded area represents the 95% CI from the restricted cubic spline model. The model is centered at the median (122 g/L) with knots at the 25th, 50th, 75th percentiles.

Among the 1682 women, 358 (21.28%) developed GDM, with the probability of developing GDM greater in those with older age, previous GDM history and higher pre-pregnancy BMI (*P* < 0.001). Compared to women with NGT, those with GDM exhibited significantly higher FBG, WBC count, neutrophil count, RBC count and Hb concentration (120 ± 11 vs. 125 ± 10 g/L in T1, 112 ± 10 vs. 115 ± 9 g/L in T2, 110 ± 12 vs. 115 ± 12 g/L in T3, all *P* < 0.001) throughout pregnancy (all *P* < 0.05), increased first-trimester blood pressure, creatinine, UA, TG, and HDL (all *P* < 0.05), second-trimester FBG, 1-h blood glucose (BG), 2-h BG, HbA1c, FINS, and HOMA-IR (all *P* < 0.001), and third-trimester diastolic blood pressure, creatinine, and TG (all *P* < 0.05). Conversely, first-trimester lymphocyte count, TC, LDL, second-trimester HOMA-β and third-trimester ALT, AST were lower in women with GDM (all *P* < 0.05) (Supplementary Table S1). Histograms revealed that women with GDM had consistently higher Hb concentrations than those with NGT throughout pregnancy (*P* < 0.001) (Fig. 2A–C).

Compared with mothers with NGT, those with GDM tended to deliver heavier newborns, had higher rates of delivering macrosomic or LGA infants, and were more likely to require cesarean sections. Composite neonatal complications and events of neonatal hypoglycemia were significantly higher in women with GDM than those with NGT (12.26% vs. 6.92%, *P* < 0.01; 3.77% vs. 0.63%, *P* < 0.001) (Supplementary Table S2). A 1:1 case-control matching procedure was performed based on age, pre-pregnancy BMI, and parity to minimize potential bias from uneven distribution of covariates. After matching, significant differences remained in RBC count and Hb concentration (NGT *vs*. GDM, 116 ± 11 vs. 123 ± 10 g/L in T1, 110 ± 11 vs. 114 ± 9 g/L in T2, 109 ± 13 vs. 115 ± 12 g/L in T3, all *P* < 0.001) and blood glucose metabolic profiles, as well as delivery and neonatal outcomes (all *P* < 0.05) (Supplementary Tables S1, S2).

### Comparison of parameters during each trimester among three groups categorized by first-trimester Hb level in the retrospective cohort study

Subjects were then divided into three groups according to Hb level in the first trimester: anemia (<110 g/L), normal Hb (≥110 and ≤130 g/L) and high Hb (>130 g/L). There was a stepwise increase in the level of pre-pregnancy BMI, diastolic blood pressure, creatinine, RBC count and Hb concentration throughout pregnancy (all *P* < 0.05), first-trimester WBC count, neutrophil count and UA (all *P* < 0.01) and second-trimester WBC count, neutrophil count and lymphocyte count (all *P* < 0.05), as well as FBG, 1-h BG, 2-h BG, HbA1c, HOMA-IR (all *P* < 0.01) and incidence of GDM (13.4%, 19.1%, and 32.1%; *P* < 0.001) across the three groups (Table 1). In contrast there was a stepwise decrease with increase Hb level in first-trimester TC and LDL (both *P* < 0.01), but no significant differences in age, previous GDM history, family history of diabetes, AST, TG, HDL, or HOMA-β among the groups (Table 1). Importantly, Pearson correlation analysis showed that first-trimester Hb level was significantly and positively correlated with FBG, 1-h BG, 2-h BG, and HOMA-IR during OGTT, but had no significant correlation with HOMA-β (Fig. 2D–G).

**Table 1:** Comparison of parameters during each trimester and at delivery among three groups categorized by first-trimester Hb level in the retrospective cohort study.

	All subjects (*n* = 1682)			
Variables	Anemia (*n* = 201)	Normal Hb (*n* = 1107)	High Hb (*n* = 374)	*P* value
First-trimester Hb (g/L)	<110	110–130	>130	
**Anthropometric parameters**				
Age (years)	27 ± 5	29 ± 10	29 ± 5	0.059
*Parity*				
Nulliparous	18 (8.95%)	189 (17.07%)	120 (32.09%)	<0.001
Parous	183 (91.04%)	918 (82.93%)	254 (67.91%)	
*Previous GDM*				
Nulliparous	18 (8.95%)	189 (17.07%)	120 (32.09%)	0.118
No	180 (89.55%)	895 (80.85%)	240 (64.17%)	
Yes	3 (1.49%)	23 (2.08%)	14 (3.74%)	
*Family history of diabetes*				
No	184 (91.54%)	1012(91.42%)	342 (91.44%)	0.566
Yes	17 (8.46%)	95 (8.58%)	32 (8.56%)	
Pre-Pregnancy BMI (kg/m^2^)	21.66 ± 2.97	22.43 ± 6.84	23.60 ± 9.79^††‡^	0.005
**First trimester (T1)**				
SBP (mmHg)	115 ± 11	116 ± 10	119 ± 11^††‡‡‡^	<0.001
DBP (mmHg)	67 ± 8	69 ± 8	73 ± 9^†††‡‡‡^	<0.001
WBCs (×10^9^/L)	8.34 ± 2.11	8.67 ± 2.01	8.97 ± 2.10^†††‡^	0.002
Neutrophils (×10^9^/L)	6.06 ± 1.75	6.32 ± 1.72^*^	6.59 ± 1.87^†††‡‡^	0.001
Lymphocytes (×10^9^/L)	1.73 ± 0.49	1.77 ± 0.49	1.80 ± 0.50	0.325
RBCs (×10^9^/L)	3.68 ± 0.50	3.96 ± 0.28^***^	4.37 ± 0.25^†††‡‡‡^	<0.001
Platelets (×10^9^/L)	221 ± 65	218 ± 52	230 ± 49^†‡‡‡^	<0.001
ALT (units/L)	9 (7–14)	12 (8–18)	13 (9–22)^†^	0.043
AST (units/L)	16 (13–18)	16 (14–19)	17 (14–22)	0.418
Cr (mmol/L)	40.07 ± 7.39	42.54 ± 7.68^***^	44.83 ± 7.88^†††‡‡‡^	<0.001
UA (umol/L)	194.80 ± 47.42	205.34 ± 42.00	217.11 ± 48.90^††‡‡^	0.001
TC (mmol/L)	5.23 ± 1.13	5.07 ± 1.08	4.87 ± 1.02^††‡^	<0.001
TG (mmol/L)	2.66 (1.94–3.32)	2.53 (2.01–3.13)	2.52 (2.03–3.41)	0.720
HDL (mmol/L)	1.63 ± 0.35	1.70 ± 0.38	1.68 ± 0.36	0.092
LDL (mmol/L)	2.98 ± 0.84	2.85 ± 0.86	2.71 ± 0.76^†††‡‡^	0.002
FBG (mmol/L)	4.33 ± 0.42	4.49 ± 0.40	4.49 ± 0.43	0.067

**Second trimester (T2)**				
OGTT time (weeks)	25.7 ± 2.2	25.7 ± 1.5	26.1 ± 1.3	0.931
*OGTT*				
FBG (mmol/L)	3.94 ± 0.58	4.10 ± 0.62^**^	4.24 ± 0.58^†††‡‡‡^	<0.001
1-h BG (mmol/L)	6.87 ± 1.80	7.25 ± 1.94^*^	8.04 ± 2.09^†††‡‡‡^	<0.001
2-h BG (mmol/L)	6.12 ± 1.29	6.48 ± 1.50^**^	6.97 ± 1.70^†††‡‡‡^	<0.001
HbA1C (%)	4.9 ± 0.3	5.0 ± 0.4^**^	5.2 ± 0.4^††‡‡^	0.003
HbA1C (mmol/mol)	30	31	33	
FINS (mIU/L)	6.50 (4.77–12.47)	8.91 (5.26–12.40)^*^	9.17 (6.01–12.71)^†††^	0.002
HOMA-IR^a^	1.34 (0.93–2.89)	1.73 (1.11–2.73)^*^	1.77 (1.16–2.73)^†††‡^	<0.001
HOMA-β^a^	162.47 (98.60–213.75)	167.17 (113.87–261.97)	180.33 (135.60–250.94)	0.531
SBP (mmHg)	114 ± 12	116 ± 10	120 ± 67	0.188
DBP (mmHg	66 ± 8	66 ± 8	68 ± 8^‡‡^	0.006
WBCs (×10^9^/L)	8.88 ± 2.16	9.48 ± 2.14^***^	9.81 ± 2.19^†††‡^	<0.001
Neutrophils (×10^9^/L)	6.38 ± 1.94	6.87 ± 1.85^**^	7.16 ± 1.87^†††‡^	<0.001
Lymphocytes (×10^9^/L)	1.80 ± 0.49	1.86 ± 0.49	1.91 ± 0.50^†^	0.032
RBCs (×10^9^/L)	3.57 ± 0.47	3.67 ± 0.26^*^	3.84 ± 0.29^†††‡‡‡^	<0.001
Hb (g/L)	103 ± 10	112 ± 8^***^	120 ± 9^†††‡‡‡^	<0.001
Platelets (×10^9^/L)	214 ± 63	210 ± 53	213 ± 50	0.531
**Third trimester (T3)**				
SBP (mmHg)	118 ± 10	120 ± 10	122 ± 11^†‡^	0.005
DBP (mmHg)	70 ± 7	73 ± 8^*^	76 ± 8^†††‡‡‡^	<0.001
WBCs (×10^9^/L)	9.40 ± 2.95	9.65 ± 2.78	9.60 ± 2.87	0.503
Neutrophils (×10^9^/L)	7.16 ± 2.80	7.33 ± 2.59	7.43 ± 4.29	0.607
Lymphocytes (×10^9^/L)	1.57 ± 0.49	1.61 ± 0.50	1.65 ± 0.54	0.123
RBCs (×10^9^/L)	3.61 ± 0.60	3.78 ± 0.33^*^	3.99 ± 0.35^†††‡‡‡^	<0.001
Hb (g/L)	103 ± 13	111 ± 11^***^	117 ± 12^†††‡‡‡^	<0.001
Platelets (×10^9^/L)	209 ± 66	207 ± 56	212 ± 53	0.349
ALT (units/L)	10 (7–14)	10 (8–14)	10 (7–14)	0.120
AST (units/L)	17 (14–21)	17 (14–20)	16 (14–20)	0.054
Creatinine (mmol/L)	45.11 ± 10.08	47.18 ± 10.29	47.84 ± 9.48^†^	0.022

UA (umol/L)	286.85 ± 70.15	292.56 ± 77.14	299.05 ± 81.46	0.714
TC (mmol/L)	5.88 ± 1.21	5.89 ± 1.25	5.59 ± 0.94	0.168
TG (mmol/L)	2.94 (2.29–3.22)	3.04 (2.32–4.00)	2.86 (2.35–3.69)	0.425
HDL (mmol/L)	1.86 ± 0.44	1.79 ± 0.39	1.76 ± 0.38	0.668
LDL (mmol/L)	3.54 ± 1.03	3.25 ± 1.23	3.13 ± 0.86	0.417
FBG (mmol/L)	4.31 ± 1.17	4.35 ± 0.53^*^	4.61 ± 0.69^†††‡‡‡^	<0.001
**Maternal outcomes**				
GDM	27 (13.4%)	211 (19.1%)	120 (32.1%)^†††‡‡‡^	<0.001
EGWG by end of pregnancy	30 (14.93%)	174 (15.72%)	65 (17.38%)	0.681
Absolute GWG (kg)	10.50 ± 4.40	11.19 ± 4.36	10.83 ± 4.66	0.586
Antenatal BMI (kg/m^2^)	26.72 ± 3.24	27.83 ± 8.72	28.29 ± 3.76	0.072
*Insulin Treatment*				
No	201 (100.00%)	1102 (99.55%)	369 (98.66%)	0.079
Yes	0 (0.00%)	5 (0.45%)	5 (1.34%)	
Hypertensive disorder of pregnancy	6 (2.99%)	85 (7.68%)^*^	30 (8.02%)^†^	0.047
**Delivery Outcomes**				
Delivery time (weeks)	39.1 ± 1.2	39.1 ± 1.3	38.7 ± 1.2	0.193
Preterm	7 (3.48%)	49 (4.43%)	13 (3.48%)	0.649
cesarean section	30 (14.93%)	236 (21.32%)^*^	95 (25.40%)^††^	0.014
*Fetus sex*				
Male	115 (57.21%)	675 (60.98%)	242 (64.71%)	0.706
Female	86 (42.79%)	432 (39.02%)	132 (35.29%)	
Birth length (cm)	49.93 ± 0.55	49.87 ± 0.96	49.81 ± 0.95	0.722
Newborn weight (g)	3375.48 ± 472.42	3363.28 ± 455.86	3372.84 ± 486.56	0.916
Macrosomia	17 (8.46%)	70 (6.32%)	28 (7.49%)	0.464
LGA	39 (19.40%)	228 (20.60%)	70 (18.72%)	0.714
SGA	13 (6.47%)	40 (3.61%)	16 (4.28%)	0.169
Apgar score <7 at 1 min	3 (1.49%)	1 (0.09%)^**^	2 (0.53%)	0.007
Apgar score <7 at 5 min	3 (1.49%)	1 (0.09%)^**^	2 (0.53%)	0.007
**Neonatal outcomes**				
Composite neonatal complications	20 (9.95%)	75 (6.78%)	41 (10.96%)^‡‡^	0.022
Neonatal hypoglycemia	3 (1.49%)	13 (1.17%)	6 (1.60%)	0.794
Hyperbilirubinemia	18 (8.96%)	65 (5.87%)	26 (6.95%)	0.241
Respiratory distress	4 (1.99%)	13 (1.17%)	9 (2.41%)	0.214
NICU admission	4 (1.99%)	13 (1.17%)	11 (2.94%)	0.065

Data are presented as mean ± SD, median (IQR), or *n* (%).

*BMI* body mass index, *SBP* systolic blood pressure, *DBP* diastolic blood pressure, *WBCs* white blood cells, *RBCs* red blood cells, *ALT* alanine aminotransferase, *AST* aspartate transaminase, *Cr* creatinine, *UA* uric acid, *TC* total cholesterol, *TG* triglyceride, *FBG* fasting blood glucose, *1-h BG* 1 h blood glucose after oral glucose tolerance test (OGTT), *2-h BG* 2 h blood glucose after OGTT, *HbA1C* glycated hemoglobin, *FINS* fasting insulin, *HOMA-IR* homeostasis model assessment of insulin resistance index, *HOMA-β* homeostasis model assessment of pancreatic β cell function index, Macrosomia was defined as birth weight >4000 g.

^a^Log-transformed for *ANOVA*.

*Normal Hb group vs. anemia group, *<0.05, **<0.01, ***<0.001.

^†^High Hb group vs. anemia group, ^††^<0.05, ^††^<0.01, ^†††^<0.001.

^‡^High Hb group vs. normal Hb group, ^‡^<0.05, ^‡‡^<0.01, ^‡‡‡^<0.001.

Regarding GDM-related maternal and delivery complications, the incidence of hypertensive disorders of pregnancy and the need for cesarean delivery increased as Hb level increased (*P* < 0.01), whereas EGWG, absolute GWG, antenatal BMI, fetus sex, neonatal weight, macrosomia, LGA, and SGA were not significantly affected (Table 1). Overall neonatal complications were significantly higher in the high Hb group than in the normal Hb group (10.96% vs. 6.78%, *P* < 0.01) (Table 1).

### Continuous Hb level in the first trimester was closely associated with the incidence of GDM

To graphically visualize the association of Hb with GDM development, a restricted cubic spline model with three knots was applied with adjustment for GDM history, maternal age and pre-pregnancy BMI. We found a significant relationship between continuous Hb level during the first trimester and GDM incidence. The risk of developing GDM increased when Hb level in the first trimester exceeded 122 g/L (Fig. 2H).

### Increased first-trimester Hb level was an independent risk factor for development of GDM

To identify independent risk factors for the development of GDM, age, pre-pregnancy BMI, increased Hb level (divided by 122 g/L), neutrophil count, platelet count, TG and creatinine in the first trimester were entered into logistic regression analysis with enter selection in all subjects without GDM history. After adjusting for potential confounding factors, higher first-trimester Hb concentration remained an independent risk factor for development of GDM (OR = 2.214, 95% CI: 1.042–4.331, *P* = 0.038) (Table 2). This relationship remained significant in the matched case-control cohort, independent of age, pre-pregnancy BMI, neutrophil count or TG level in the first trimester (OR = 4.968, 95% CI: 2.480–9.954, *P* < 0.001) (Table 2). Additionally, combining Hb concentration with basal factors (age, pre-pregnancy BMI, TG, and FBG in the first trimester) yielded the highest area under the receiver operating characteristic curve (AUC) for predicting GDM. This combination achieved an AUC of 0.795, outperforming basal factors alone (0.786), as well as combinations with other first-trimester blood cell indicators such as neutrophil count (0.787) and platelet count (0.786). Corresponding sensitivities and specificities for these combinations were 0.783 and 0.746 for Hb with basal factors, 0.696 and 0.815 for basal factors alone, 0.710 and 0.821 for neutrophil count with basal factors, and 0.710 and 0.785 for platelet count with basal factors, respectively (Supplementary Fig. S2).

**Table 2:** Logistic regression analysis to determine the risk factors for development of GDM in all subjects without GDM history and in the retrospective case-control study.

	In all subjects without GDM history (*n* = 1642)		In matched case-control study (*n* = 620)	
	OR (95% CI)	*P*	OR (95% CI)	*P*
Age (years)	1.103 (1.027–1.185)	<0.01	0.941 (0.856–1.032)	0.445
Pre-Pregnancy BMI (kg/m^2^)	1.112 (1.001–1.236)	0.049	0.983 (0.890–1.085)	0.730
*Hb group in T1*				
Hb ≤122 g/L	Reference		Reference	
Hb >122 g/L	2.124 (1.042–4.331)	0.038	4.968 (2.480–9.954)	<0.001
Neutrophils in T1 (×10^9^/L)	1.304 (1.079–1.576)	0.006	1.388 (1.154–1.669)	<0.001
Platelets in T1 (×10^9^/L)	0.989 (0.981–0.996)	0.004	0.998 (0.992–1.004)	0.512
TG in T1 (mmol/L)	1.371 (1.098–1.713)	0.005	1.307 (1.062–1.609)	0.011
Creatinine in T1 (mmol/L)	1.054 (1.011–1.100)	0.014	1.020 (0.982–1.060)	0.312

### Women with Hp2-2 genotype combined with elevated first-trimester Hb concentration were at higher risk of GDM development

**Fig. 3:**
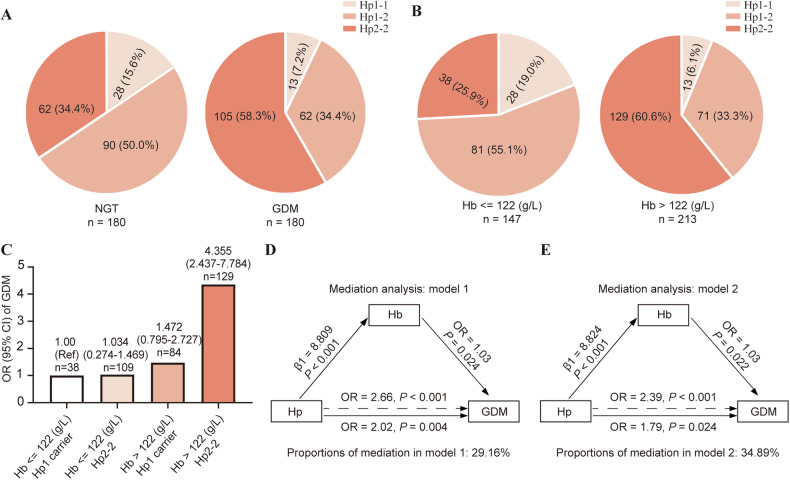
Distribution, joint and mediation analysis of Hp phenotypes and Hb levels in relation to GDM risk. Frequency distribution of Hp phenotypes in women with NGT and with GDM (**A**) and in Hb >122 g/L group versus Hb ≤122 g/L group (**B**) in the validation cohort study. Multivariate OR of GDM risk according to Hb status and Hp genotype (**C**). Mediation analysis of first-trimester Hb level and Hp genotype on GDM risk without (**D**) or with (**E**) adjusting for confounding factors, age and pre-pregnancy BMI.

To investigate the moderating effect of Hp genotype on the association between Hb levels and GDM, Hp genotypes were determined in 180 women with NGT and 180 women with GDM, who were 1:1 matched based on pre-pregnancy BMI, age, and parity from a pool of 400 subjects. The proportion of genotype Hp1-1, Hp1-2, and Hp2-2 was 15.6%, 50.0%, and 34.4%, respectively, in healthy controls, close to the reported frequency in China. Nonetheless, the frequency of the Hp1-1 genotype in women with GDM was much lower than in healthy controls while the frequency of Hp2-2 was much higher (*P* < 0.001) (Fig. 3A). Compared to those with Hp1 carrier genotype (both Hp1-1 and Hp1-2 genotypes), women with the Hp2-2 genotype had much higher RBC count and Hb concentration throughout pregnancy (all *P* < 0.001). They also had higher first-trimester blood pressure and creatinine, second-trimester FBG, 1-h BG, 2-h BG, HbA1c, FINS, HOMA-IR, and GDM incidence, as well as third-trimester FBG (all *P* < 0.05) (Supplementary Table S3). Additionally, overall neonatal complications were more frequent in women with the Hp2-2 genotype (*P* < 0.05) (Supplementary Table S4). Meanwhile, the frequency of Hp1-1 and Hp1-2 genotypes was significantly lower in women with Hb > 122 g/L than in those with Hb ≤ 122 g/L (4.69% *vs*. 10.94%, 30.21% vs. 46.09%, *P* < 0.001), whereas the frequency of Hp2-2 was much higher (65.10% vs. 42.97%, *P* < 0.001) (Fig. 3B). After adjusting for potential confounding factors, logistic regression analysis showed that both higher first-trimester Hb concentration and the HP2-2 genotype remained independent risk factors for the development of GDM (Supplementary Table S5).

To identify independent risk factors for the development of GDM, age, pre-pregnancy BMI, increased Hb level (divided by 122 g/L), neutrophil count, platelet count, TG and creatinine in the first trimester were entered into logistic regression analysis with enter selection in all subjects without GDM history. After adjusting for potential confounding factors, higher first-trimester Hb concentration remained an independent risk factor for the development of GDM (OR = 2.214, 95% CI: 1.042–4.331, *P* = 0.038) (Table 2).

### Interaction moderate effect, joint analysis, and mediation analysis of first-trimester Hb level and Hp genotype with GDM risk

After adjusting for age and pre-pregnancy BMI, a significant multiplicative interaction effect was observed between Hp genotype and first-trimester Hb level on GDM development (OR = 4.801, 95% CI:1.705–13.517, *P* for interaction = 0.003) (Table 3). Further analysis revealed a statistically significant synergistic stimulation additive interaction effect of first-trimester Hb level and Hp genotype on the occurrence of GDM (RERI = 3.472, 95% CI: 1.629–6.697) (Table 3).

**Table 3:** Interaction analysis of Hp genotype and Hb level for GDM risk.

Group	Hp1 carrier	Hp2–2	Effect of Hp genotype stratified by Hb
Hb ≤122 g/L	1 [Reference]	0.655 (0.283–1.516)	0.655 (0.283–1.516)
Hb >122 g/L	1.459 (0.791–2.690)	4.587 (2.572–8.179)	3.144 (1.717–5.755)
Effect of Hb stratified by Hp genotype	1.459 (0.791–2.690)	7.005 (3.034–16.174)	
Multiplicative scale	4.801 (1.705–13.517)		
RERI	3.472 (1.629–6.697)		
AP	0.757 (0.425–0.936)		
SI	31.542 (1.001–789089)		

*RERI* Relative excess risk due to interaction, *AP* Attributable proportion due to interaction, *SI* the synergy index.

To explore the joint associations of first-trimester Hb level and Hp genotype with GDM risk, pregnant women were stratified according to their Hb level and Hp genotype. As shown in Fig. 3C, after adjusting for age and pre-pregnancy BMI, the OR of risk for GDM development increased in a stepwise manner from women with Hb ≤ 122 g/L and Hp1 allele to women with Hb > 122 g/L and Hp2-2 genotype (OR = 1.034, 95% CI: 0.274–1.469, *P* = 0.288; OR = 1.472, 95% CI: 0.795–2.727, *P* = 0.219; OR = 4.355, 95% CI: 2.437–7.784, *P* < 0.001; respectively).

Mediation analysis was performed to evaluate the influence of first-trimester Hb level and Hp genotype on GDM risk (Fig. 3D, E). Hp genotype was found to be an independent risk factor for GDM without adjusting for confounding factors (OR = 2.66, 95% CI: 1.74–4.10), and it remained an independent risk factor for GDM after adjusting for age and pre-pregnancy BMI (OR = 2.39, 95% CI: 1.54–3.75). Additionally, first-trimester Hb level partially mediated the association of Hp genotype with GDM risk, explaining 29.16% of the association without adjusting for other factors (Fig. 3D). After adjusting for age and pre-pregnancy BMI, first-trimester Hb level explained 34.89% of the association (Fig. 3E).

## Discussion

First, our study confirms the association between high Hb levels and GDM development in a combined retrospective case-control and cohort study with large sample size. Elevated Hb levels have raised concerns regarding the increased risk of adverse maternal and neonatal outcomes. Recent evidence indicates that high Hb levels and biomarkers indicating elevated iron stores are associated with a risk of developing GDM [4, 6, 22], though previous studies primarily focus on Hb levels during the first and/or second trimester of pregnancy. We established that Hb concentrations are increased throughout pregnancy in women with GDM. Consequently, first-trimester Hb levels hold significant potential as early diagnostic markers for GDM and play a critical role in its pathogenesis. In the cohort study, further analysis demonstrated a progressive increase in GDM incidence and insulin resistance with rising first-trimester Hb levels. A fully adjusted spline regression showed a significant correlation of continuous first-trimester Hb levels with GDM incidence, with the risk abruptly increasing when Hb level exceeded 122 g/L. In addition, the role of high Hb level as an independent risk factor for GDM development was confirmed in all subjects and in the case-control study.

Another important finding of this study is the interaction between Hb levels and Haptoglobin (Hp) genotype concerning GDM risk. Oxidative stress is increasingly recognized as a pivotal factor in GDM [23, 24]. The primary function of the Hp protein is to bind Hb during hemolysis, thereby reducing Fe^2+^ deposition in the body. The Hp-Hb complex is rapidly cleared from the bloodstream by CD163 scavenger receptors expressed in monocytes/macrophages. Different Hp alleles vary in their ability to clear free Hb from the plasma, with Hp2-2-Hb complexes being less efficiently cleared than non-Hp2-2-Hb complexes [13]. Consequently, subjects with the Hp2-2 genotype are more prone to oxidative stress [25], potentially influencing Hb concentrations in pregnant women. To validate this hypothesis, we explored the interplay between high Hb concentrations and Hp genotype in relation to GDM risk. Consistently, the frequency of Hp2-2 was much higher in women with GDM than those with NGT, and in women with Hb >122 g/L than in women with Hb ≤122 g/L, suggesting that Hp1-1 genotype protects against higher Hb and GDM development. Moreover, our study is the first to demonstrate a significant additive interaction between the Hp2-2 genotype and Hb levels above 122 g/L in relation to GDM occurrence. Additionally, this study is the first to show that first-trimester Hb levels partially mediated the association of Hp genotype with GDM risk. This suggests that the Hp2-2 genotype’s role in GDM development is connected to its function in scavenging free Hb, indicating that targeting elevated Hb concentrations may be a viable therapeutic strategy for GDM prevention, tailored to Hp polymorphism.

Our study also confirmed that GDM is associated with adverse pregnancy outcomes, including macrosomia, LGA, the need for cesarean section and neonatal hypoglycemia, similar to previous reports [3]. Likewise, the incidence of hypertensive disorders of pregnancy, cesarean delivery and overall adverse neonatal outcomes were significantly increased as Hb level increased. Recent studies have shown that maternal Hb level has a positive correlation with hypertension in preeclampsia [26, 27]. Studies of the influence of maternal Hb on delivery mode and neonatal outcomes are limited and sometimes contradictory; maternal anemia in pregnancy usually represents a common and potentially reversible risk factor associated with perinatal complications [28]. Our results demonstrate that increased Hb concentrations contribute more significantly to composite neonatal outcomes.

There are some limitations to this study. First, all subjects were recruited from The Fifth People’s Hospital of Shanghai and Wujing Hospital and may limit the generalizability of our findings. Second, we could not evaluate the exposure-response effect of dietary iron and folate intake on Hb concentration, GDM, and adverse pregnancy outcomes, which is critical for reducing these risks. We also acknowledge that mechanistic insights into the potential pathophysiological role of high Hb level in combination with the Hp2-2 genotype in GDM development are lacking in this clinical study. Further studies using reliable rodent GDM models to delineate the function of Hp are warranted.

## Conclusions

This study demonstrated that increased Hb concentration was closely associated with GDM development as well as maternal and neonatal outcomes. Specifically, first-trimester Hb concentration was identified as an independent risk factor for GDM, with a significant linear association observed when Hb exceeded 122 g/L. Furthermore, our findings suggest that targeted screening for the Hp polymorphism among individuals with Hb >122 g/L could help identify those who are at high risk of GDM. Such individuals could benefit more from individualized iron supplementation strategies, potentially reducing the risk of hyperglycemia and subsequent adverse pregnancy outcomes.

### Supplementary information

Supplementary file

## Data Availability

The data sets generated and/or analyzed during the current study are not publicly available but may be obtained from the corresponding author upon reasonable request.
