# Supplementary material for: First-trimester hemoglobin, haptoglobin genotype, and risk of gestational diabetes mellitus in a retrospective study among Chinese pregnant women
Source: Nutr Diabetes. 2024 Jun 29;14:48. doi: 10.1038/s41387-024-00309-y (PMC11217379; doi:10.1038/s41387-024-00309-y)
Supplement: Supplementary file 1 — Supplementary file [file 41387_2024_309_MOESM1_ESM.docx]

Online-Only Supplemental Material

Methods

Hp Genotyping

The PCR protocol for Hp genotyping was as follows: Two-step PCR was performed; the first PCR reaction contained 25 µL of Taq MIX polymerase, 1.5 µL of primer A (5-GAGGGGAGCTTGCCTTTCCATTG-3), 1.5 µL of primer B (5-GAGATTTTTGAGCCCTGGCTGGT-3), 15 µL of DNA, and 7 µL of ddH2O. The second PCR reaction contained 25 µL of Taq MIX polymerase, 1.5 µL of primer C (5-CCTGCCTCGTATTAACTGCACCAT-3), 1.5 µL of primer D (5-CCGAGTGCTCCACATAGCCATGT-3), 10 µL of DNA, and 12 µL of ddH2O. After an initial denaturation step at 94℃ for 3 min, the three-step thermocycling procedure consisted of denaturation at 94℃ for 30 s and annealing for 30 s, and extension for 4 min at 72℃ (in the presence of primers A and B) or 1 min (in the presence of primers C and D only) for 35 cycles followed by a final extension at 72℃ for 5 min.

Results

Legends

Supplementary Fig. S1. Electropherogram with Hp phenotype patterns, as observed in blood samples.

Supplementary Fig. S2. Prediction of GDM with the ROC curves by Hb concentration, neutrophil count or platelet count combined with basal factors (age, pre-pregnancy BMI, TG and FBG in first trimester)

Supplementary Table S1. Characteristics of women with and without GDM in all subjects and in the retrospective case-control study. Data are presented as mean ± SD, median (IQR), or n (%).

Supplementary Table S2. Maternal, delivery and neonatal outcomes of women with and without GDM in all subjects and in the retrospective case-control study. Data are presented as mean ± SD, median (IQR), or n (%).

Supplementary Table S3. Comparison of parameters during each trimester between two groups categorized by Hp genotypes in the validation cohort study. Data are presented as mean ± SD, median (IQR), or n (%).

Supplementary Table S4. Comparison of maternal, delivery and neonatal outcomes between two groups categorized by Hp genotypes in the validation cohort study. Data are presented as mean ± SD, median (IQR), or n (%).

Supplementary Table S5. Logistic regression analysis to determine the risk factors for development of GDM in the validation cohort study.

Supplementary Fig. S1. Electropherogram with Hp phenotype patterns, as observed in blood samples. Primer A and B: lanes 1-8, Primer C and D: lanes 9-16 from blood samples of 8 patients same as lanes 1-8, respectively; Hp phenotypes: Hp1-1 (lanes 2/10, 6/14), Hp1-2 (lanes 1/9, 4/12, 7/15), Hp2-2 (lanes 3/11, 5/13, 8/16).

**
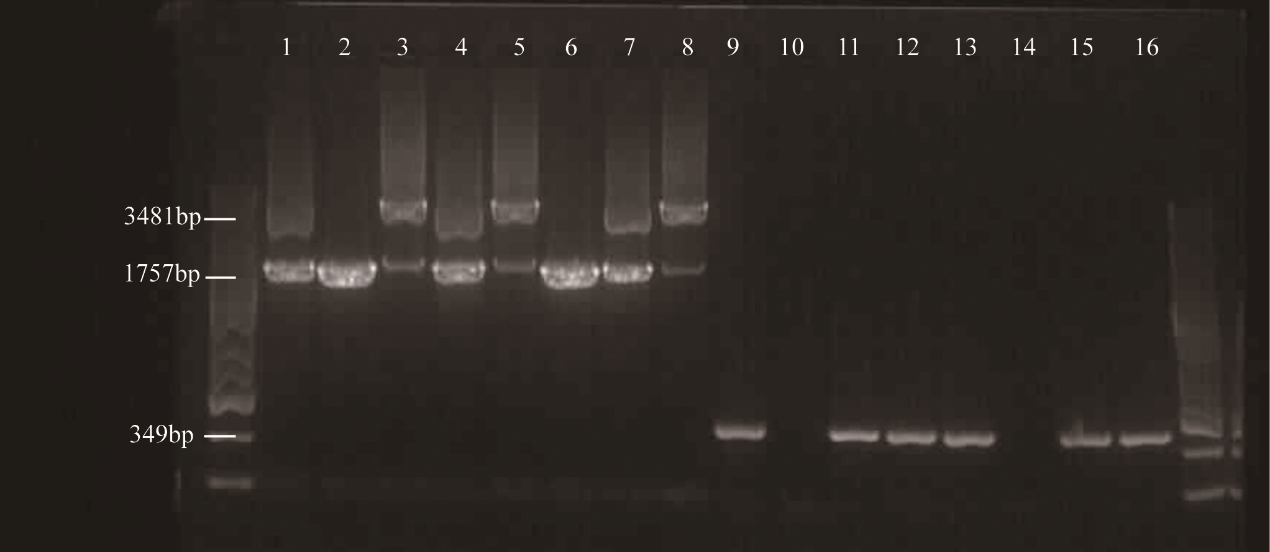
**

Supplementary Fig. S2. Prediction of GDM with the ROC curves by Hb concentration, neutrophil count or platelet count combined with basal factors (age, pre-pregnancy BMI, TG and FBG in first trimester)

**
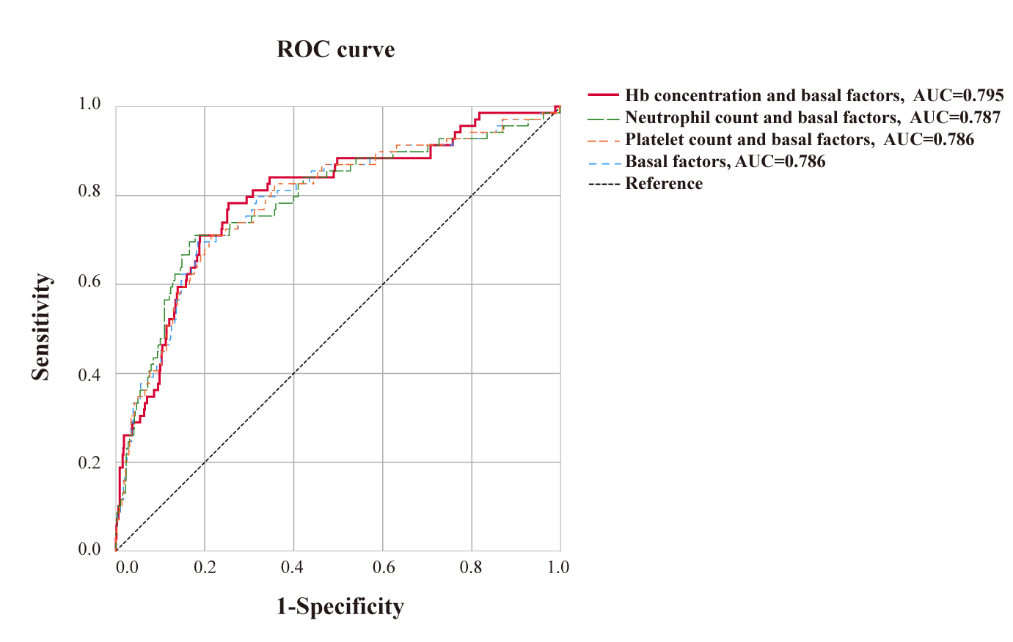
**

Supplementary Table S1. Characteristics of women with and without GDM in all subjects and in the retrospective case-control study

|  | **All subjects (n = 1682)** | | | |  | **Matched case-control study (n = 620)** | | | |
| --- | --- | --- | --- | --- | --- | --- | --- | --- | --- |
| **Variables** | **Total** | **Con** | **GDM** | ***P* value** |  | **Total** | **Con** | **GDM** | ***P* value** |
|  | **(n = 1682)** | **(n = 1324)** | **(n = 358)** |  |  | **(n = 620)** | **(n = 310)** | **(n = 310)** |  |
| **Anthropometric parameter** |  |  |  |  |  |  |  |  |  |
| Age (years) | 28.51 ± 8.91 | 27.87 ± 9.63 | 30.91 ± 4.71 | <0.001 |  | 30 ± 4 | 30 ± 4 | 30 ± 4 | 0.239 |
| Parity |  |  |  |  |  |  |  |  |  |
| Nulliparous | 327 (19.44%) | 200 (15.11%) | 127 (35.47%) | <0.001 |  | 178 (28.71%) | 89 (28.71%) | 89 (28.71%) | 1.000 |
| Parous | 1355 (80.56%) | 1124 (84.89%) | 231 (64.53%) |  |  | 442 (71.29%) | 221 (71.29%) | 221 (71.29%) |  |
| Previous GDM |  |  |  |  |  |  |  |  |  |
| Nulliparous | 327 (19.44%) | 200 (15.11%) | 127 (35.47%) | <0.001 |  | 178 (28.71%) | 89 (28.71%) | 89 (28.71%) | <0.001 |
| No | 1315 (78.18%) | 1124 (84.89%) | 191 (53.35%) |  |  | 400 (64.52%) | 221 (71.29%) | 179 (57.74%) |  |
| Yes | 40 (2.38%) | 0 (0.00%) | 40 (11.17%) |  |  | 42 (6.77%) | 0 (0.00%) | 42 (13.55%) |  |
| Family history of diabetes |  |  |  |  |  |  |  |  |  |
| No | 1538 (91.44%) | 1212 (91.54%) | 326 (91.06%) | 0.155 |  | 567 (91.45%) | 284 (91.61%) | 283 (91.29%) | 0.539 |
| Yes | 144 (8.56%) | 112 (8.46%) | 32 (8.94%) |  |  | 53 (8.55%) | 26 (8.39%) | 27 (8.71%) |  |
| Pre-Pregnancy BMI (kg/m^2^) | 22.60 ± 7.30 | 22.10 ± 3.13 | 24.50 ± 14.69 | <0.001 |  | 22.91 ± 2.96 | 22.91 ± 2.96 | 22.91 ± 2.97 | 0.991 |
| **First trimester (T1)** |  |  |  |  |  |  |  |  |  |
| SBP (mmHg) | 117 ± 10 | 116 ± 10 | 118 ± 11 | 0.006 |  | 117 ± 10 | 117 ± 10 | 118 ± 11 | 0.396 |
| DBP (mmHg) | 70 ± 8 | 70 ± 8 | 71 ± 9 | 0.009 |  | 70 ± 9 | 69 ± 8 | 71 ± 9 | 0.116 |
| WBCs (× 10^9^/L) | 8.70 ± 2.04 | 8.61 ± 2.02 | 9.04 ± 2.10 | <0.001 |  | 8.75 ± 2.14 | 8.40 ± 2.00 | 9.11 ± 2.22 | <0.001 |
| Neutrophils (× 10^9^/L) | 6.35 ± 1.76 | 6.24 ± 1.73 | 6.75 ± 1.85 | <0.001 |  | 6.47 ± 1.87 | 6.14 ± 1.75 | 6.80 ± 1.93 | <0.001 |
| Lymphocytes (× 10^9^/L) | 1.77 ± 0.49 | 1.79 ± 0.50 | 1.72 ± 0.47 | 0.013 |  | 1.71 ± 0.47 | 1.69 ± 0.46 | 1.73 ± 0.48 | 0.381 |
| RBCs (× 10^9^/L) | 4.07 ± 0.36 | 4.02 ± 0.35 | 4.10 ± 0.37 | 0.014 |  | 4.06 ± 0.38 | 4.03 ± 0.40 | 4.08 ± 0.38 | 0.267 |
| Hb (g/L) | 122 ± 11 | 120 ± 11 | 125 ± 10 | <0.001 |  | 120 ± 12 | 116 ± 11 | 123 ± 10 | <0.001 |
| Platelets (× 10^9^/L) | 221 ± 53 | 218 ± 53 | 231 ± 54 | <0.001 |  | 224 ± 57 | 217 ± 56 | 230 ± 56 | 0.007 |
| ALT (units/L) | 12 (8-18) | 12 (9-19) | 12 (8-18) | 0.162 |  | 13 (9-21) | 14 (10-22) | 12 (9-20) | 0.030 |
| AST (units/L) | 16 (14-20) | 16 (14-20) | 16 (14-20) | 0.051 |  | 17 (14-21) | 17 (14-22) | 17 (14-20) | 0.048 |
| Cr (mmol/L) | 42.77 ± 7.81 | 41.98 ± 7.47 | 45.67 ± 8.32 | <0.001 |  | 43.82 ± 8.45 | 42.22 ± 7.93 | 45.39 ± 8.65 | <0.001 |
| UA (umol/L) | 208.43 ± 45.02 | 204.93 ± 42.48 | 213.90 ± 48.31 | 0.012 |  | 211.63 ± 47.52 | 207.69 ± 44.15 | 213.50 ± 49.03 | 0.312 |
| TC (mmol/L) | 5.04 ± 1.08 | 5.12 ± 1.07 | 4.81 ± 1.07 | <0.001 |  | 4.95 ± 1.06 | 5.10 ± 1.03 | 4.83 ± 1.07 | 0.003 |
| TG (mmol/L) | 2.54 (2.01-3.20) | 2.50 (1.97-3.12) | 2.94 (2.38-3.65) | <0.001 |  | 2.12 (2.76-3.58) | 2.62 (2.04-3.18) | 3.00 (2.46-3.78) | 0.002 |
| HDL (mmol/L) | 1.69 ± 0.37 | 1.68 ± 0.38 | 1.73 ± 0.34 | 0.032 |  | 1.7 ± 0.36 | 1.68 ± 0.37 | 1.71 ± 0.35 | 0.331 |
| LDL (mmol/L) | 2.83 ± 0.84 | 2.88 ± 0.83 | 2.68 ± 0.83 | <0.001 |  | 2.75 ± 0.83 | 2.85 ± 0.79 | 2.67 ± 0.84 | 0.012 |
| **FBG (mmol/L)** | 4.47 ±0.41 | 4.42 ± 0.36 | 4.55 ± 0.48 | <0.001 |  | 4.51 ± 0.45 | 4.45 ± 0.36 | 4.64 ± 0.48 | 0.017 |
| **Second trimester (T2)** |  |  |  |  |  |  |  |  |  |
| OGTT time (weeks) | 25.7 ± 1.5 | 25.7 ± 1.4 | 25.8 ± 1.7 | 0.813 |  | 25.8 ± 1.7 | 25.6 ± 1.4 | 26.0 ± 2.1 | 0.250 |
| OGTT |  |  |  |  |  |  |  |  |  |
| FBG (mmol/L) | 4.11 ± 0.61 | 3.97 ± 0.49 | 4.66 ± 0.72 | <0.001 |  | 4.32 ± 0.64 | 4.04 ± 0.49 | 4.61 ± 0.64 | <0.001 |
| 1-h BG (mmol/L) | 7.38 ± 1.99 | 6.67 ± 1.41 | 9.93 ± 1.66 | <0.001 |  | 8.37 ± 2.18 | 6.77 ± 1.39 | 9.91 ± 1.62 | <0.001 |
| 2-h BG (mmol/L) | 6.55 ± 1.55 | 6.01 ± 1.04 | 8.37 ± 1.57 | <0.001 |  | 7.31 ± 1.72 | 6.10 ± 1.06 | 8.37 ± 1.47 | <0.001 |
| HbA1C (%) | 5.0 ± 0.4 | 4.9 ± 0.3 | 5.2 ± 0.4 | <0.001 |  | 5.1 ± 0.4 | 4.9 ± 0.3 | 5.2 ± 0.4 | <0.001 |
| HbA1C (mmol/mol) | 31 | 30 | 33 |  |  | 32 | 30 | 33 |  |
| FINS (mIU/L) | 8.85 (5.44-12.53) | 6.77 (4.94-9.32) | 10.14 (6.44-13.81) | <0.001 |  | 9.23 (6.20-13.12) | 7.21 (5.50-10.62) | 9.73 (7.14-13.69) | <0.001 |
| HOMA-IR^§^ | 1.72 (1.12-2.73) | 1.28 (0.91-1.86) | 2.15 (1.30-3.08) | <0.001 |  | 1.73 (1.15-2.73) | 1.25 (0.94-1.91) | 1.94 (1.35-2.92) | <0.001 |
| HOMA-β^§^ | 172.71 (115.91-247.97) | 182.74 (118.27-281.01) | 163.93 (115.35-235.67) | <0.001 |  | 183.14 (113.07-339.17) | 220.00 (64.38-478.15) | 179.57 (117.89-309.35) | <0.001 |
| **SBP (mmHg)** | 117 ± 36 | 117 ± 42 | 116 ± 10 | 0.834 |  | 116 ± 10 | 116 ± 10 | 116 ± 10 | 0.881 |
| **DBP (mmHg)** | 67 ± 8 | 67 ± 8 | 66 ± 8 | 0.135 |  | 66 ± 8 | 67 ± 8 | 66 ± 8 | 0.140 |
| **WBCs (×10^9^/L)** | 9.48 ± 2.17 | 9.42 ± 2.19 | 9.71 ± 2.10 | 0.030 |  | 9.38 ± 2.18 | 9.09 ± 2.14 | 9.66 ± 2.18 | <0.001 |
| **Neutrophils (×10^9^/L)** | 6.88 ± 1.88 | 6.80 ± 1.89 | 7.17 ± 1.81 | <0.001 |  | 6.85 ± 1.86 | 6.58 ± 1.85 | 7.12 ± 1.84 | <0.001 |
| **Lymphocytes (×10^9^/L)** | 1.87 ± 0.49 | 1.88 ± 0.50 | 1.82 ± 0.47 | 0.031 |  | 1.82 ± 0.47 | 1.81 ± 0.46 | 1.82 ± 0.48 | 0.854 |
| **RBCs (×109/L)** | 3.65 ± 0.31 | 3.63 ± 0.30 | 3.69 ± 0.31 | 0.024 |  | 3.65 ± 0.31 | 3.62 ± 0.33 | 3.68 ± 0.31 | 0.027 |
| **Hb (g/L)** | 113 ± 10 | 112 ± 10 | 115 ± 9 | <0.001 |  | 112 ± 10 | 110 ± 11 | 114 ± 9 | <0.001 |
| **Platelets (×10^9^/L)** | 211 ± 54 | 211 ± 55 | 214 ± 51 | 0.392 |  | 211 ± 54 | 210 ± 56 | 212 ± 52 | 0.684 |
| **Third trimester (T3)** |  |  |  |  |  |  |  |  |  |
| **SBP (mmHg)** | 120 ± 10 | 119 ± 10 | 121 ± 10 | 0.055 |  | 120 ± 10 | 119 ± 10 | 120 ± 10 | 0.700 |
| **DBP (mmHg)** | 73 ± 8 | 72 ± 8 | 75 ± 8 | <0.001 |  | 73 ± 8 | 72 ± 8 | 74 ± 8 | 0.018 |
| **WBCs (×10^9^/L)** | 9.61 ± 2.82 | 9.05 ± 2.64 | 9.76 ± 2.85 | <0.001 |  | 9.22 ± 2.6 | 9.25 ± 2.45 | 9.19 ± 2.74 | 0.760 |
| **Neutrophils (×10^9^/L)** | 7.33 ± 3.06 | 6.94 ± 4.17 | 7.44 ± 2.68 | 0.008 |  | 6.96 ± 2.41 | 7.03 ± 2.31 | 6.88 ± 2.51 | 0.432 |
| **Lymphocytes (×10^9^/L)** | 1.61 ± 0.51 | 1.62 ± 0.51 | 1.59 ± 0.49 | 0.481 |  | 1.55 ± 0.5 | 1.51 ± 0.48 | 1.6 ± 0.51 | 0.027 |
| **RBCs (×109/L)** | 3.83 ± 0.37 | 3.80 ± 0.36 | 3.86 ± 0.40 | 0.019 |  | 3.82 ± 0.39 | 3.80 ± 0.36 | 3.85 ± 0.40 | 0.018 |
| **Hb (g/L)** | 111 ± 13 | 110 ± 12 | 115 ± 12 | <0.001 |  | 112 ± 13 | 109 ± 13 | 115 ± 12 | <0.001 |
| **Platelets (×10^9^/L)** | 208 ± 57 | 209 ± 57 | 204 ± 56 | 0.198 |  | 205 ± 58 | 206 ± 58 | 205 ± 59 | 0.860 |
| **ALT (units/L)** | 10 (7-14) | 10 (8-14) | 10 (6-14) | 0.047 |  | 10 (7-15) | 8 (5-10) | 8 (6-12) | 0.469 |
| **AST (units/L)** | 17 (14-20) | 17 (15-20) | 15 (12-18) | <0.001 |  | 16 (14-20) | 16 (13-19) | 15 (13-18) | <0.001 |
| **Creatinine (mmol/L)** | 47.04 ± 10.12 | 46.26 ± 9.53 | 50.32 ± 11.73 | <0.001 |  | 48.66 ± 10.83 | 47.32 ± 10.14 | 50.12 ± 11.38 | 0.007 |
| **UA (umol/L)** | 294.33 ± 78.03 | 293.52 ± 78.36 | 295.33 ± 77.88 | 0.835 |  | 290.47 ± 73.25 | 277.59 ± 72.42 | 295.65 ± 73.24 | 0.146 |
| **TC (mmol/L)** | 5.79 ± 1.16 | 5.76 ± 1.04 | 5.83 ± 1.28 | 0.660 |  | 5.77 ± 1.23 | 5.61 ± 0.98 | 5.85 ± 1.32 | 0.293 |
| **TG (mmol/L)** | 3.00 (2.33-3.87) | 2.72 (2.25-3.46) | 3.16 (2.47-4.07) | <0.001 |  | 3.06 (2.40-4.01) | 2.92 (2.34-3.55) | 3.20 (2.49-4.11) | 0.055 |
| **HDL (mmol/L)** | 1.78 ± 0.39 | 1.77 ± 0.36 | 1.80 ± 0.42 | 0.505 |  | 1.78 ± 0.41 | 1.75 ± 0.33 | 1.79 ± 0.44 | 0.538 |
| **LDL (mmol/L)** | 3.23 ± 1.11 | 3.31 ± 0.96 | 3.13 ± 1.25 | 0.197 |  | 3.19 ± 1.18 | 3.21 ± 0.89 | 3.18 ± 1.29 | 0.907 |
| **FBG (mmol/L)** | 4.33 ± 0.64 | 4.18 ± 0.44 | 4.44 ± 0.74 | 0.004 |  | 4.47 ± 0.67 | 4.38 ± 0.39 | 4.67 ± 0.73 | 0.013 |

Data are presented as mean ± SD, median (IQR), or n (%). Abbreviation: NA, not applicable.

Supplementary Table S2. Maternal, delivery and neonatal outcomes of women with and without GDM in all subjects and in the retrospective case-control study

|  | **All subjects (n = 1682)** | | | |  | **Matched case-control study (n = 620)** | | | |
| --- | --- | --- | --- | --- | --- | --- | --- | --- | --- |
| **Variable** | **Total** | **Con** | **GDM** | ***P* value** |  | **Total** | **Con** | **GDM** | ***P* value** |
|  | **(n = 1682)** | **(n = 1324)** | **(n = 358)** |  |  | **(n = 620)** | **(n = 310)** | **(n = 310)** |  |
| **Maternal outcomes** |  |  |  |  |  |  |  |  |  |
| EGWG by end of pregnancy | 269 (16.00%) | 225 (16.99%) | 44 (12.16%) | 0.031 |  | 68 (10.97%) | 33 (10.53%) | 35 (11.17%) | 0.797 |
| Absolute GWG (kg) | 13.39 ± 4.88 | 13.78 ± 4.95 | 11.97 ± 4.36 | <0.001 |  | 12.82 ± 4.45 | 13.38 ± 4.65 | 12.30 ± 4.20 | 0.004 |
| Antenatal BMI (kg/m^2^) | 27.78 ± 7.42 | 27.48 ± 3.41 | 28.86 ± 14.38 | 0.003 |  | 27.91 ± 3.13 | 28.15 ± 3.17 | 27.69 ± 3.08 | 0.088 |
| Treatment |  |  |  |  |  |  |  |  |  |
| Lifestyle intervention | NA | NA | 348 (97.21%) | NA |  | NA | NA | 302 (97.41%) | NA |
| Insulin | NA | NA | 10 (2.79%) |  |  | NA | NA | 8 (2.58%) |  |
| Hypertensive disorders of pregnancy | 121 (7.19%) | 87 (6.56%) | 34 (9.62%) | 0.057 |  | 39 (6.29%) | 16 (5.13%) | 23 (7.28%) | 0.247 |
| **Delivery Outcomes** |  |  |  |  |  |  |  |  |  |
| Delivery time (weeks) | 38.9 ± 1.3 | 39.0 ± 1.3 | 38.7 ± 1.1 | 0.041 |  | 39.2 ± 1.2 | 39.2 ± 1.3 | 38.7 ± 1.2 | 0.030 |
| Preterm | 69 (4.10%) | 49 (3.72%) | 20 (5.63%) | 0.11 |  | 31 (5.00%) | 13 (4.27%) | 18 (5.92%) | 0.357 |
| Cesarean section | 361 (21.46%) | 247 (18.66%) | 114 (31.84%) | <0.001 |  | 158 (25.48%) | 58 (18.71%) | 100 (32.26%) | <0.001 |
| Fetus sex |  |  |  |  |  |  |  |  |  |
| Male | 837 (49.76%) | 667 (50.38%) | 170 (47.49%) | 0.712 |  | 390 (62.90%) | 197 (63.55%) | 193 (62.26%) | 0.595 |
| Female | 845 (50.24%) | 657 (49.62%) | 188 (52.51%) |  |  | 230 (37.10%) | 113 (36.45%) | 117 (37.74%) |  |
| Newborn length (cm) | 49.85 ± 0.94 | 49.8 ± 1.0 | 49.9 ± 0.8 | 0.316 |  | 49.9 ± 1.0 | 49.9 ± 1.3 | 49.9 ± 0.9 | 0.454 |
| Newborn weight (g) | 3366.83 ± 464.28 | 3356.26 ± 459.37 | 3409.19 ± 481.94 | 0.079 |  | 3422.50 ± 496.75 | 3434.64 ± 496.67 | 3410.13 ± 497.45 | 0.566 |
| Macrosomia | 115 (6.84%) | 82 (6.19%) | 33 (9.22%) | 0.044 |  | 65 (10.48%) | 32 (10.32%) | 33 (10.65%) | 0.896 |
| LGA | 337 (20.03%) | 251 (18.98%) | 86 (24.00%) | 0.034 |  | 148 (23.87%) | 73 (23.53%) | 75 (24.26%) | 0.851 |
| SGA | 69 (4.10%) | 53 (4.01%) | 16 (4.50%) | 0.693 |  | 29 (4.68%) | 18 (5.88%) | 11 (3.55%) | 0.183 |
| Apgar score <7 at 1 min | 6 (0.36%) | 4 (0.31%) | 2 (0.47%) | 0.470 |  | 6 (0.97%) | 4 (1.23%) | 2 (0.58%) | 0.682 |
| Apgar score <7 at 5 min | 6 (0.36%) | 4 (0.31%) | 2 (0.47%) | 0.470 |  | 6 (0.97%) | 4 (1.23%) | 2 (0.58%) | 0.682 |
| **Neonatal outcomes** |  |  |  |  |  |  |  |  |  |
| Composite neonatal complications | 136 (8.09%) | 92 (6.92%) | 44 (12.26%) | 0.001 |  | 46 (7.42%) | 16 (5.16%) | 30 (9.83%) | 0.031 |
| Neonatal hypoglycemia | 22 (1.31%) | 8 (0.63%) | 14 (3.77%) | <0.001 |  | 14 (2.26%) | 3 (0.97%) | 11 (3.47%) | 0.031 |
| Hyperbilirubinemia | 109 (6.48%) | 79 (5.97%) | 30 (8.49%) | 0.100 |  | 37 (5.97%) | 15 (4.84%) | 22 (6.94%) | 0.235 |
| Respiratory distress | 26 (1.55%) | 21 (1.57%) | 5 (1.42%) | 0.797 |  | 7 (1.13%) | 3 (0.97%) | 4 (1.23%) | 0.704 |
| NICU admission | 28 (1.66%) | 21 (1.57%) | 7 (1.89%) | 0.628 |  | 7 (1.13%) | 3 (0.97%) | 4 (1.23%) | 0.704 |

Supplementary Table S3. Comparison of parameters during each trimester between two groups categorized by Hp genotypes in the validation cohort study

| **Variables** | **Total** | **Hp1 carrier** | **Hp2-2** | ***P* value** |
| --- | --- | --- | --- | --- |
|  | **(n = 360)** | **(n = 193)** | **(n = 167)** |  |
| **Anthropometric parameters** |  |  |  |  |
| Age (years) | 30 ± 4 | 30 ± 4 | 31 ± 5 | 0.079 |
| Parity |  |  |  |  |
| Nulliparous | 160 (44.44%) | 88 (45.60%) | 72 (43.11%) | 0.636 |
| Parous | 200 (55.56%) | 105 (54.40%) | 95 (56.89%) |  |
| Previous GDM |  |  |  |  |
| Nulliparous | 160 (44.44%) | 88 (45.60%) | 72 (43.11%) | 0.714 |
| No | 197 (54.73%) | 104 (53.89%) | 93 (55.69%) |  |
| Yes | 3 (0.83%) | 1 (0.68%) | 2 (0.94%) |  |
| Family history of diabetes |  |  |  |  |
| No | 329 (91.39%) | 134 (91.16%) | 195 (91.55%) | 0.279 |
| Yes | 31 (8.61%) | 13 (8.84%) | 18 (8.45%) |  |
| Pre-Pregnancy BMI (kg/m^2^) | 22.80 ± 9.76 | 21.93 ± 3.03 | 23.79 ± 13.84 | 0.073 |
| **First trimester (T1)** |  |  |  |  |
| SBP (mmHg) | 117 ± 11 | 116 ± 11 | 118 ± 11 | 0.037 |
| DBP (mmHg) | 70 ± 8 | 69 ± 8 | 72 ± 8 | <0.001 |
| WBCs (×10^9^/L) | 8.89 ± 1.95 | 8.94 ± 2.02 | 8.84 ± 1.86 | 0.639 |
| Neutrophils (×10^9^/L) | 6.7 ± 1.7 | 6.75 ± 1.75 | 6.65 ± 1.65 | 0.589 |
| Lymphocytes (×10^9^/L) | 1.63 ± 0.42 | 1.63 ± 0.41 | 1.63 ± 0.43 | 1.000 |
| RBCs (×109/L) | 4.09 ± 0.37 | 3.93 ± 0.33 | 4.28 ± 0.32 | <0.001 |
| Hb (g/L) | 124 ± 10 | 120 ± 8 | 130 ± 10 | <0.001 |
| Platelets (×10^9^/L) | 229 ± 51 | 228 ± 49 | 229 ± 54 | 0.956 |
| ALT (units/L) | 12 (9-19) | 12 (8-18) | 12 (9-21) | 0.197 |
| AST (units/L) | 17 (14-20) | 17 (14-20) | 17 (14-22) | 0.549 |
| Cr (mmol/L) | 47.12 ± 7.54 | 46.36 ± 6.76 | 47.99 ± 8.27 | 0.041 |
| UA (umol/L) | 206.26 ± 46.45 | 202.81 ± 47.20 | 210.31 ± 45.37 | 0.145 |
| TC (mmol/L) | 4.67 ± 0.91 | 4.69 ± 0.85 | 4.65 ± 0.98 | 0.638 |
| TG (mmol/L) | 1.54 (1.25-1.96) | 1.50 (1.24-2.00) | 1.58 (1.30-1.86) | 0.743 |
| HDL (mmol/L) | 1.81 ± 0.36 | 1.86 ± 0.36 | 1.76 ± 0.35 | 0.018 |
| LDL (mmol/L) | 2.60 ± 0.72 | 2.59 ± 0.7 | 2.61 ± 0.74 | 0.891 |
| FBG (mmol/L) | 4.49 ± 0.45 | 4.46 ± 0.42 | 4.53 ± 0.48 | 0.194 |
| **Second trimester (T2)** |  |  |  |  |
| OGTT time (weeks) | 25.7 ± 1.3 | 26.1 ± 1.5 | 25.7 ± 1.2 | 0.735 |
| OGTT |  |  |  |  |
| FBG (mmol/L) | 4.34 ± 0.59 | 4.00 ± 0.59 | 4.58 ± 0.60 | <0.001 |
| 1-h BG (mmol/L) | 8.55 ± 2.16 | 8.15 ± 2.16 | 9.02 ± 2.06 | <0.001 |
| 2-h BG (mmol/L) | 7.35 ± 1.77 | 7.03 ± 1.76 | 7.72 ± 1.71 | <0.001 |
| HbA1C (%) | 5.1 ± 0.4 | 5.0 ± 0.4 | 5.2 ± 0.3 | <0.001 |
| HbA1C (mmol/mol) | 32 | 31 | 33 |  |
| FINS (mIU/L) | 9.03 (5.63-12.60) | 8.16 (5.12-11.27) | 9.62 (6.59-13.03) | 0.017 |
| HOMA-IR^§^ | 1.70 (1.15-2.71) | 1.63 (0.99-2.59) | 1.88 (1.19-2.81) | 0.025 |
| HOMA-β^§^ | 180.96 (123.22-269.78) | 169.71 (114.62-277.00) | 188.00 (135.76-264.74) | 0.250 |
| SBP (mmHg) | 114 ± 10 | 114 ± 10 | 115 ± 10 | 0.278 |
| DBP (mmHg | 65 ± 8 | 65 ± 8 | 66 ± 8 | 0.221 |
| WBCs (×10^9^/L) | 9.64 ± 2.12 | 9.65 ± 2.24 | 9.61 ± 1.98 | 0.861 |
| Neutrophils (×10^9^/L) | 7.12 ± 1.80 | 7.11 ± 1.87 | 7.13 ± 1.71 | 0.939 |
| Lymphocytes (×10^9^/L) | 1.80 ± 0.45 | 1.82 ± 0.44 | 1.78 ± 0.46 | 0.379 |
| RBCs (×109/L) | 3.67 ± 0.31 | 3.58 ± 0.28 | 3.78 ± 0.30 | <0.001 |
| Hb (g/L) | 115 ± 9 | 111 ± 8 | 119 ± 8 | <0.001 |
| Platelets (×10^9^/L) | 212 ± 49 | 213 ± 48 | 211 ± 50 | 0.758 |
| **Third trimester (T3)** |  |  |  |  |
| SBP (mmHg) | 120 ± 11 | 119 ± 10 | 121 ± 11 | 0.075 |
| DBP (mmHg) | 75 ± 8 | 74 ± 8 | 76 ± 8 | 0.145 |
| WBCs (×10^9^/L) | 9.00 ± 2.45 | 9.11 ± 2.42 | 8.87 ± 2.49 | 0.365 |
| Neutrophils (×10^9^/L) | 6.70 ± 2.21 | 6.82 ± 2.15 | 6.55 ± 2.27 | 0.267 |
| Lymphocytes (×10^9^/L) | 1.60 ± 0.48 | 1.59 ± 0.43 | 1.62 ± 0.52 | 0.564 |
| RBCs (×109/L) | 3.83 ± 0.36 | 3.75 ± 0.33 | 3.92 ± 0.37 | <0.001 |
| Hb (g/L) | 116 ± 11 | 114 ± 10 | 119 ± 11 | <0.001 |
| Platelets (×10^9^/L) | 208 ± 54 | 206 ± 54 | 210 ± 54 | 0.452 |
| ALT (units/L) | 8 (6-12) | 9 (7-12) | 8 (6-12) | 0.404 |
| AST (units/L) | 15 (13-18) | 16 (13-18) | 15 (13-18) | 0.126 |
| Creatinine (mmol/L) | 51.59 ± 9.61 | 51.82 ± 9.83 | 51.32 ± 9.39 | 0.700 |
| UA (umol/L) | 292.67 ± 80.03 | 292.07 ± 86.51 | 293.37 ± 72.07 | 0.908 |
| TC (mmol/L) | 5.80 ± 1.18 | 5.87 ± 1.08 | 5.74 ± 1.29 | 0.473 |
| TG (mmol/L) | 3.00 (2.33-3.66) | 3.02 (2.25-3.75) | 2.98 (2.40-3.66) | 0.665 |
| HDL (mmol/L) | 1.81 ± 0.40 | 1.84 ± 0.38 | 1.77 ± 0.42 | 0.211 |
| LDL (mmol/L) | 3.26 ± 1.15 | 3.28 ± 1.04 | 3.24 ± 1.27 | 0.843 |
| FBG (mmol/L) | 4.36 ± 0.64 | 4.04 ± 0.69 | 4.50 ± 0.58 | 0.003 |

Data are presented as mean ± SD, median (IQR), or n (%).

Supplementary Table S4. Comparison of maternal, delivery and neonatal outcomes between two groups categorized by Hp genotypes in the validation cohort study.

| **Variables** | **Total** | **Hp1 carrier** | **Hp2-2** | ***P* value** |
| --- | --- | --- | --- | --- |
|  | **(n = 360)** | **(n = 193)** | **(n = 167)** |  |
| **Maternal outcomes** |  |  |  |  |
| GDM |  |  |  |  |
| No | 180 (50.00%) | 98 (66.67%) | 82 (38.50%) | <0.001 |
| Yes | 180 (50.00%) | 49 (33.33%) | 131 (61.50%) |  |
| EGWG by end of pregnancy | 36 (10.00%) | 18 (9.33%) | 18 (10.78%) | 0.457 |
| Absolute GWG (kg) | 11.12 ± 4.29 | 11.15 ± 4.28 | 11.08 ± 4.33 | 0.884 |
| Antenatal BMI (kg/m^2^) | 27.42 ± 3.50 | 27.18 ± 3.33 | 27.72 ± 3.69 | 0.161 |
| Treatment |  |  |  |  |
| Lifestyle intervention | 129 (35.83%) | 46 (23.83%) | 83 (49.70%) | 0.105  0.318 |
| Insulin | 38 (10.56%) | 18 (9.33%) | 20 (11.98%) |  |
| Hypertensive disorders of pregnancy | 15 (4.17%) | 6 (3.11%) | 9 (5.39%) | 0.214 |
| **Delivery Outcomes** |  |  |  |  |
| Delivery time (weeks) | 39.1 ± 1.2 | 39.0 ± 1.2 | 39.1 ± 1.2 | 0.010 |
| Preterm | 22 (6.11%) | 12 (6.22%) | 10 (5.99%) | 0.908 |
| Cesarean section | 131 (36.39%) | 70 (36.27%) | 61 (36.53%) | 0.965 |
| Fetus sex |  |  |  |  |
| Male | 220 (61.11%) | 124 (64.25%) | 106 (63.47%) | 0.901 |
| Female | 140 (38.89%) | 69 (42.86%) | 61 (36.53%) |  |
| Birth length (cm) | 49.84 ± 0.99 | 49.86 ± 1.04 | 49.83 ± 0.94 | 0.849 |
| Newborn weight (g) | 3358.57 ± 470.09 | 3349.84 ± 460.67 | 3368.31 ± 481.85 | 0.737 |
| Macrosomia | 29 (8.06%) | 15 (7.77%) | 14 (8.38%) | 0.926 |
| LGA | 73 (20.28%) | 37 (19.17%) | 36 (21.56%) | 0.665 |
| SGA | 17 (4.72%) | 8 (4.15%) | 9 (5.39%) | 0.553 |
| Apgar score <7 at 1 min | 2 (0.55%) | 0 (0.00%) | 2 (1.20%) | 0.414 |
| Apgar score <7 at 5 min | 0 (0.00%) | 0 (0.00%) | 0 (0.00%) | NA |
| **Neonatal outcomes** |  |  |  |  |
| Composite neonatal complications | 39 (10.00%) | 16 (8.29%) | 20 (11.98%) | 0.049 |
| Neonatal hypoglycemia | 9 (2.50%) | 4 (2.07%) | 5 (2.99%) | 0.358 |
| Hyperbilirubinemia | 29 (8.06%) | 14 (7.25%) | 15 (8.98%) | 0.119 |
| Respiratory distress | 3 (0.83%) | 0 (0.00%) | 3 (1.80%) | 0.101 |
| NICU admission | 3 (0.83%) | 0 (0.00%) | 3 (1.80%) | 0.101 |

Supplementary Table S5. Logistic regression analysis to determine the risk factors for development of GDM in the validation cohort study.

| **Variables** | **Model 1** | |  | **Model 2** | |
| --- | --- | --- | --- | --- | --- |
|  | **OR (95% CI)** | ***P*** |  | **OR (95% CI)** | ***P*** |
| Hb group in T1 |  |  |  |  |  |
| Hb ≤122 g/L | Reference |  |  |  |  |
| Hb >122 g/L | 3.513 (2.169-5.691) | <0.001 |  |  |  |
| Hp genotype |  |  |  |  |  |
| Hp1-1 |  |  |  | Reference |  |
| Hp1-2 |  |  |  | 1.233 (0.566-2.687) | 0.598 |
| Hp2-2 |  |  |  | 2.871 (1.322-6.236) | 0.008 |
| Age (years) | 1.059 (0.997-1.124) | 0.063 |  | 0.941 (0.856-1.032) | 0.445 |
| Pre-Pregnancy BMI (kg/m^2^) | 1.035 (0.944-1.134) | 0.467 |  | 0.983 (0.890-1.085) | 0.730 |
| Neutrophils in T1 (×10^9^/L) | 1.176 (1.022-1.352) | 0.023 |  | 1.388 (1.154-1.669) | <0.001 |
| Platelets in T1 (×10^9^/L) | 1.000 (0.995-1.004) | 0.936 |  | 0.998 (0.992-1.004) | 0.512 |
| TG in T1 (mmol/L) | 1.443 (1.074-1.939) | 0.015 |  | 1.307 (1.062-1.609) | 0.011 |
| Creatinine in T1 (mmol/L) | 0.993 (0.963-1.024) | 0.658 |  | 1.020 (0.982-1.060) | 0.312 |
